# Prospective Research Comparing Different Trochanteric Fracture Fixation Techniques

**DOI:** 10.7759/cureus.67774

**Published:** 2024-08-25

**Authors:** Swaroop Solunke, Abhishek Nair, Rahul Agrawal, Ashwin Deshmukh, Ankit Barosani

**Affiliations:** 1 Orthopaedics, Dr D Y Patil Medical College Hospital and Research Center, Dr D Y Patil Vidyapeeth (Deemed to be University) Pimpri, Pune, IND

**Keywords:** rehabilitation, proximal femoral nail, harris hip score, fixation, trochanteric fracture

## Abstract

Aim

Evaluation and comparison of various methods of trochanteric fracture fixation.

Methods

This study was conducted prospectively at the Orthopaedics Department of Dr. D Y Patil Medical College and Research Centre. The study spanned 18 months and involved 100 patients treated in the outpatient and the emergency department. Patients who fulfilled that specific criteria were selected for this study and the appropriate surgical intervention for each group was determined through radiological examination.

Results

Of the 100 patients, 55 (55%) were male and 45 (45%) females. Patients in our collection ranged from 20 to 90 years old. Proximal Femoral Nail (PFN), Dynamic Hip Screw (DHS ), and Proximal Femoral Locking Compression Plate (PFLCP ) groups averaged 56, 58, and 64 years old, respectively. The most common cause of intertrochanteric fracture was domestic falls (60%), followed by road traffic accidents (35%). The Arbeitsgemeinschaft für Osteosynthesefragen (AO) classification rated 40 individuals (40%) as stable and 60 as unstable. Most patients in our study had unstable A3 fractures. PFN patients had 16 A3 fractures. In DHS, 32 patients suffered A3 fractures. Twelve PFLCP patients suffered A3 fractures. The smallest group had A1 fractures. Six PFN patients suffered A1 fractures. Two DHS patients had A1 fractures. Two PFLCP patients suffered A1 fractures. PFN group mean scores improved significantly after one and three months in this study. At six months, the PFN group had a significant mean score improvement.

Conclusion

PFN results ranged from satisfactory to excellent, offering numerous advantages over other methods such as DHS and PFLCP. The benefits of PFN include a shorter lever arm, fracture site compression, and enhanced rotational stability, which contribute to a lower chance of mechanical failure. Additionally, patients treated with PFN typically experience shorter hospital stays, earlier mobilization, less blood loss, shorter surgery times, faster rehabilitation, and quicker bone healing, making PFN a highly effective treatment option for certain fractures.

## Introduction

The incidence of hip fractures is on the rise as the population continues to age globally. It is estimated that by 2040, there will be a staggering 512,000 cases of hip fractures [1]. Early mobilization and minimizing complications are possible through surgical treatment using stable fixation. Two methods are used to fix trochanteric fractures: plate fixation and intramedullary implants [2,3]. The dynamic hip screw (DHS), also known as the sliding hip screw (SHS), is a commonly used implant for treating trochanteric fractures [4-6]. However, DHS has a biomechanical drawback compared to intramedullary implants due to a larger gap between the weight-bearing axis and the implants [7].

The proximal femoral nail (PFN) has become increasingly popular for treating trochanteric fractures. Since its introduction in 1998, PFN has gained significant recognition [8-11]. Numerous studies have highlighted the benefits of the proximal femoral nail, including better outcomes in terms of stability and recovery [12-13]. However, PFN is still associated with occasional technical issues [14]. Additionally, the cost of PFN is significantly greater than that of DHS, making cost a consideration in treatment decisions.

Various methods are used for treating unstable trochanteric fractures, including intramedullary nails, sliding plate devices, proximal femoral locking plates, angular blade plates, and trochanteric stabilization plates [14,15]. Sliding plate devices can result in inward movement of the lower fragment and excessive collapse of the upper fragment in unstable intertrochanteric fractures with lateral wall comminution and implant failure [16]. Implants such as PFN are advantageous as they reduce the bending force exerted upon the implant by minimizing the lever arm and being positioned closer to the mechanical axis of the extremity [17]. Placement of the PFN within the bone helps distribute the load, preventing the lower fragment from shifting inward and the upper fragment from experiencing excessive compression. This method shortens surgery time, reduces blood loss, and allows the patient to start bearing weight sooner. Additionally, it leads to decreased long-term shortening during the follow-up period [18,11].

## Materials and methods

Study design and participation criteria

This observational study was conducted over eighteen months at the orthopedic department's outpatient clinic and emergency room. One hundred patients were recruited based on predetermined criteria. Eligibility included patients over 18 with closed intertrochanteric fractures sustained within two weeks before enrollment and those willing to provide informed consent for a minimum follow-up period of 12 months. Exclusion criteria were pathological fractures, compound fractures, polytrauma, comorbidities such as paralytic limbs, and patients undergoing chemotherapy or radiotherapy. The study was approved by the institutional ethics committee (IESC/W/160/2024).

Data collection

Demographic data, including age, gender, and mechanism of injury, were collected. The fracture type was classified according to the Arbeitsgemeinschaft für Osteosynthesefragen (AO) classification system. Patients were then divided into three groups based on the fixation technique used which included PFN, DHS, and proximal femoral locking compression plate (PFLCP).

Outcome measures

The primary outcome measure was the Harris Hip Score (HHS) assessed at one, three, six, and 12 months postoperatively. Secondary outcomes included fracture healing time, complications, and the need for reoperation.

Surgical procedures

All surgeries were performed by experienced orthopedic surgeons under similar conditions. The specific techniques for each fixation method are described below.

For the PFN procedure, the patient was positioned supine on a fracture table. A longitudinal incision was made along the lateral aspect of the thigh. A guidewire was inserted through the tip of the greater trochanter, followed by the reaming of the canal. The PFN was then inserted, and proximal and distal screws were placed to ensure stabilization.

In the DHS procedure, the patient was also positioned supine on a fracture table. A lateral incision was made over the proximal femur. A guidewire was inserted through the lateral cortex into the femoral head. A sliding hip screw and side plate were applied, followed by the insertion of cortical screws to secure the fixation.

For the PFLCP procedure, the patient was positioned supine on a fracture table. A longitudinal incision was made over the lateral aspect of the thigh. The fracture was reduced and temporarily fixed with K-wires. The PFLCP was then applied along the lateral aspect of the femur and secured with locking screws.

Postoperative care

Postoperative care was standardized for all patients to ensure consistency in recovery. This included antibiotic prophylaxis for 24 hours to prevent infection. Thromboprophylaxis was administered using low molecular weight heparin to reduce the risk of deep vein thrombosis. Pain management was achieved with oral analgesics. Early mobilization was encouraged, with weight-bearing as tolerated to promote faster rehabilitation and recovery.

Follow-up and assessment

Postoperative follow-up included regular monitoring at four-week intervals for the first three months, then every three months for one year. Clinical and radiological assessments were conducted at these intervals. Outcomes were evaluated using the Harris Hip Scoring System at one month, three months, six months, and 12 months. This scoring system assesses hip function, with scores classified as excellent (90-100), good (80-89), fair (70-79), and poor (below 70).

Statistical analysis

Statistical analysis was performed using SPSS (IBM Corp. Released 2011. IBM SPSS Statistics for Windows, Version 20.0. Armonk, NY: IBM Corp). The objective was to determine significant differences between groups. A P value of less than 0.05 was considered statistically significant.

## Results

The demographic data showed a nearly equal distribution of males (55%) and females (45%) among the study population. Patients receiving the PFLCP were generally older, with a mean age of 64 years, compared to those undergoing PFN (mean age 56 years) and DHS(mean age 58 years) procedures. This suggests that the choice of fixation device may correlate with patient age, potentially reflecting the different clinical considerations or severity of conditions treated in older versus younger patients (Table 1).

**Table 1:** Demographic parameters

Gender	N%
Males	55 (55)
Females	45 (45)
Mean age among groups	
Proximal femoral nail	56
Dynamic Hip Screw	58
Proximal Femoral Locking Compression Plate	64

Types of injuries leading to hip fixation procedures and the specific procedures utilized, including PFN, DHS, and PFLCP. Domestic falls are the most prevalent cause of injury, accounting for 60% of the cases, with 25 patients treated with PFN, 24 with DHS, and 11 with PFLCP. Road traffic accidents (RTA) are the second most common cause, representing 35% of the cases, with 16 patients receiving PFN, 10 undergoing DHS, and nine treated with PFLCP. Assaults constitute the smallest group, at 5%, with three patients treated with PFN, two with DHS, and no cases requiring PFLCP (Table 2).

**Table 2:** Type of injury and corresponding procedure The table describes the types of injuries leading to hip fixation procedures and the specific procedures utilized. PFN: proximal femoral nail, DHS: dynamic hip screw, PFLCP: proximal femoral locking compression plate

Injury type	Procedure			
	PFN	DHS	PFLCP	Total (%)
Road traffic accidents	16	10	9	35 (35)
Domestic fall	25	24	11	60 (60)
Assault	3	2	-	5 (5)

According to the AO classification, 40% of the patients were categorized as stable, while 60% were unstable. The investigation revealed that most patients had A3-type unstable fractures. The PFN group consisted of 16 patients, the DHS group 32 patients, and the PFLCP group consisted of 12 patients. All patients in these groups had A3-type fractures. The number of patients with A1-type fractures was the lowest. Among the patients in our study, six were in the PFN group, two were in the DHS group, and two were in the PFLCP group, all of whom had A1-type fractures (Table 3).

**Table 3:** AO classification of fracture and fracture pattern The table shows the distribution of patients based on the AO classification and fracture pattern within each type of fracture. AO: Arbeitsgemeinschaft für Osteosynthesefragen, PFN: proximal femoral nail, DHS: dynamic hip screw, PFLCP: proximal femoral locking compression plate.

AO classification	PFN	DHS	PFLCP
A1	6	2	2
A2	6	22	2
A3	16	32	12
Fracture pattern			
Stable (40)	12	24	4
Unstable (60)	16	32	12

PFN patients showed notable recovery, with overall scores increasing from 44.5 at one month to 92.8 at 12 months, and both stable and unstable fractures achieved high scores by the end. DHS patients also exhibited substantial progress, with scores rising from 37.8 at one month to 92.3 at 12 months, and stable fractures maintaining a slight advantage over unstable ones. PFLCP patients, although starting with lower scores, demonstrated steady improvement from 37.2 at one month to 87.8 at 12 months, with stable fractures showing better early recovery but converging with unstable fractures over time. These findings highlight the effectiveness of all three procedures in enhancing hip function, with the greatest long-term benefits seen in PFN and DHS patients (Table 4).

**Table 4:** Evaluation of Harris hip score for the patient groups at 1, 3, 6, and 12 months follow-up PFN: proximal femoral nail, DHS: dynamic hip screw, PFLCP: proximal femoral locking compression plate.

Group and classification	1 Month	3 Months	6 Months	12 Months
PFN	44.5	72.8	88.3	92.8
Stable	46.2	76.2	87.9	87.1
Unstable	43.7	67.8	69.2	91.9
DHS (B)	37.8	62.2	83.5	92.3
Stable	39.7	61.9	84.8	92.3
Unstable	36.9	58.8	82.1	88.2
PFLCP (C)	37.2	54.2	77.2	87.8
Stable	46.8	57.1	76.4	89.5
Unstable	35.6	52.9	74.7	87.0

## Discussion

Intertrochanteric fractures are common, particularly among older individuals with weakened bones, often resulting from minor accidents like simple falls [19]. The occurrence of intertrochanteric fractures varies globally. Predictions by Gulberg et al. indicate a significant increase in hip fractures, expected to reach 2.6 million by 2025 [20]. Internal fixation is preferred for fractures in the intertrochanteric femur, with DHS, PFN, and PFLCP commonly used.

Among the 100 patients, we found that the most frequent cause of intertrochanteric fractures was the domestic fall, accounting for 60 (60%) cases. Fractures from road traffic accidents were the second most common cause, making up 35% of cases. Based on the AO classification, 40 (40%) patients were found stable, while the remaining 60% were unstable. In a study by Gadegone et al., 2017 [21], patients with unstable trochanteric femoral fractures underwent a procedure where a PFN was used to fix the fractures. All cases showed bone healing within 14.2 weeks, according to their report. Complications were observed in nine patients, which included distal interlocking bolt failure, the Z-effect, lateral migration of neck screws, and infection. In another study on unstable intertrochanteric fractures, Huang et al., 2020 found that using cerclage cable in addition to PFN resulted in better fracture stabilization and superior outcomes [22]. These included higher Harris Hip Scores, faster fracture healing, weight-bearing, and reduced postoperative complications. Patients also reported improved self-care.

Unstable fractures of A3 type were the most common type of fracture in our study while A1 type fractures were least frequent. In the present study, there was a significant improvement in the mean score at one month and three months in the PFN group. After six months, the average score in the PFN group showed significant improvement. However, after one year of the follow-up, the Harris Hip score revealed no significant difference in the functional status in any of the groups. The study conducted by Kulkarni et al. 2017 found that utilizing cerclage wires and lag screws for lateral wall reconstruction can effectively reduce complications and lead to positive radiological and functional results in unstable trochanteric fractures [23]. In another recent study, Purushotham et al., 2020 found that adding an extra screw or cerclage wire to the proximal femoral nail can greatly enhance its effectiveness and stability in treating unstable intertrochanteric fractures [24]. This improvement in the construct not only promotes faster healing but also reduces the time required for union.

The comprehensive evaluation of different trochanteric fracture fixation techniques in this study provided valuable insights into the efficacy and outcomes of each method. Including the patient population with demographic variability and a robust follow-up period further enhances the reliability of the findings. However, limitations include the relatively small sample size and a single-center study, which may affect the generalizability of the results. Additionally, the absence of long-term follow-up data, makes it difficult to assess the durability and long-term outcomes of the fixation techniques.

## Conclusions

The PFN offers exceptional rotational stability, effectively compresses the fracture site, and reduces the length of the lever arm. Consequently, there is a decline in the occurrence of mechanical malfunction, a drop in the duration of hospitalization, an earlier initiation of movement, a reduction in the amount of blood loss, and a decrease in the duration of surgical procedures. In addition, PFN enables early recovery and accelerates healing compared to DHS and PFLCP. The DHS Proximal femoral locking plate is a suitable choice for certain cases of complicated, fragmented, and unstable intertrochanteric fractures in persons with osteoporosis. The plate securely stabilizes the fracture in the aligned position, as defined by the surgeon, without requiring controlled collapse.

## References

[1] Cummings SR, Rubin SM, Black D (1990). The future of hip fractures in the United States. Numbers, costs, and potential effects of postmenopausal estrogen. Clin Orthop.

[2] Utrilla AL, Reig JS, Muñoz FM, Tufanisco CB (2005). Trochanteric gamma nail and compression hip screw for trochanteric fractures: a randomized, prospective, comparative study in 210 elderly patients with a new design of the gamma nail. J Orthop Trauma.

[3] Parker MJ, Handoll HH (2008). Gamma and other cephalocondylic intramedullary nails versus extramedullary implants for extracapsular hip fractures in adults. Cochrane Database Syst Rev.

[4] Butt MS, Krikler SJ, Nafie S, Ali MS (1995). Comparison of dynamic hip screw and gamma nail: a prospective, randomized, controlled trial. Injury.

[5] Bridle SH, Patel AD, Bircher M, Calvert PT (1991). Fixation of intertrochanteric fractures of the femur. A randomised prospective comparison of the gamma nail and the dynamic hip screw. J Bone Joint Surg Br.

[6] Goldhagen PR, O'Connor DR, Schwarze D, Schwartz E (1994). A prospective comparative study of the compression hip screw and the gamma nail. J Orthop Trauma.

[7] Holz U (2015). The elements of fracture fixation. Indian J Orthop.

[8] Banan H, Al-Sabti A, Jimulia T, Hart AJ (2002). The treatment of unstable, extracapsular hip fractures with the AO/ASIF proximal femoral nail (PFN)-our first 60 cases. Injury.

[9] Schipper IB, Bresina S, Wahl D, Linke B, Van Vugt AB, Schneider E (2002). Biomechanical evaluation of the proximal femoral nail. Clin Orthop Relat Res.

[10] Al-yassari G, Langstaff RJ, Jones JWM, Al-Lami M (2002). The AO/ASIF proximal femoral nail (PFN) for the treatment of unstable trochanteric femoral fracture. Injury.

[11] Gadegone WM, Salphale YS (2007). Proximal femoral nail - an analysis of 100 cases of proximal femoral fractures with an average follow up of 1 year. Int Orthop.

[12] Nuber S, Schönweiss T, Rüter A (2003). Stabilisation of unstable trochanteric femoral fractures. Dynamic hip screw (DHS) with trochanteric stabilisation plate vs. proximal femur nail (PFN) (Article in German). Unfallchirurg.

[13] Pajarinen J, Lindahl J, Michelsson O, Savolainen V, Hirvensalo E (2005). Pertrochanteric femoral fractures treated with a dynamic hip screw or a proximal femoral nail. A randomised study comparing post-operative rehabilitation. J Bone Joint Surg Br.

[14] Pavelka T, Matejka J, Cervenková H (2005). Complications of internal fixation by a short proximal femoral nail. Acta Chir Orthop Traumatol Cech.

[15] Selim AA, Beder FK, Algeaidy IT, Farhat AS, Diab NM, Barakat AS (2020). Management of unstable pertrochanteric fractures, evaluation of forgotten treatment options. SICOT J.

[16] Medoff RJ, Maes K (1991). A new device for the fixation of unstable pertrochanteric fractures of the hip. J Bone Joint Surg Am.

[17] Walmsley D, Nicayenzi B, Kuzyk PR, Machin A, Bougherara H, Schemitsch EH, Zdero R (2016). Biomechanical analysis of the cephalomedullary nail versus the trochanteric stabilizing plate for unstable intertrochanteric femur fractures. Proc Inst Mech Eng H.

[18] Watson ST, Schaller TM, Tanner SL, Adams JD, Jeray KJ (2016). Outcomes of low-energy basicervical proximal femoral fractures treated with cephalomedullary fixation. J Bone Joint Surg Am.

[19] Dimon JH, Hughston JC (1967). Unstable intertrochanteric fractures of the hip. J Bone Joint Surg Am.

[20] Gullberg B, Johnell O, Kanis JA (1997). World-wide projections for hip fracture. Osteoporos Int.

[21] Gadegone WM, Shivashankar B, Lokhande V, Salphale Y (2017). Augmentation of proximal femoral nail in unstable trochanteric fractures. SICOT J.

[22] Huang C, Wu X (2021). Surgical selection of unstable intertrochanteric fractures: PFNA combined with or without cerclage cable. Biomed Res Int.

[23] Kulkarni SG, Babhulkar SS, Kulkarni SM, Kulkarni GS, Kulkarni MS, Patil R (2017). Augmentation of intramedullary nailing in unstable intertrochanteric fractures using cerclage wire and lag screws: a comparative study. Injury.

[24] J PV, Khan MA, Kumar N (2020). Efficacy of proximal femoral nail augmentation in unstable intertrochanteric fracture. Int J Res Orthop.

